# Improving stable isotope assessments of inter‐ and intra‐species variation in coral reef fish trophic strategies

**DOI:** 10.1002/ece3.9221

**Published:** 2022-09-13

**Authors:** Jonathan D. Cybulski, Christina Skinner, Zhongyue Wan, Carmen K. M. Wong, Robert J. Toonen, Michelle R. Gaither, Keryea Soong, Alex S. J. Wyatt, David M. Baker

**Affiliations:** ^1^ The Swire Institute of Marine Science The University of Hong Kong Shek O Hong Kong SAR; ^2^ School of Biological Sciences The University of Hong Kong Pok Fu Lam Hong Kong SAR; ^3^ Department of Ocean Science The Hong Kong University of Science and Technology Clear Water Bay Hong Kong SAR; ^4^ State Key Laboratory of Marine Pollution City University of Hong Kong Kowloon Hong Kong SAR; ^5^ Hawai‘i Institute of Marine Biology, School of Ocean & Earth Sciences & Technology University of Hawai‘i at Mānoa Kaneohe Hawaii USA; ^6^ Department of Biology University of Central Florida Orlando Florida USA; ^7^ Department of Oceanography National Sun Yat‐sen University Kaohsiung Taiwan

**Keywords:** individual specialization, isotopic niche, reef fish diets, SIBER, stable isotope analysis, trophic ecology, trophodynamics, δ15N, δ13C, and δ34S

## Abstract

Fish have one of the highest occurrences of individual specialization in trophic strategies among Eukaryotes. Yet, few studies characterize this variation during trophic niche analysis, limiting our understanding of aquatic food web dynamics. Stable isotope analysis (SIA) with advanced Bayesian statistics is one way to incorporate this individual trophic variation when quantifying niche size. However, studies using SIA to investigate trophodynamics have mostly focused on species‐ or guild‐level (i.e., assumed similar trophic strategy) analyses in settings where source isotopes are well‐resolved. These parameters are uncommon in an ecological context. Here, we use Stable Isotope Bayesian Ellipses in R (SIBER) to investigate cross‐guild trophodynamics of 11 reef fish species within an oceanic atoll. We compared two‐ (*δ* ^15^N and *δ* ^13^C) versus three‐dimensional (*δ* ^15^N, *δ* ^13^C, and *δ* ^34^S) reconstructions of isotopic niche space for interpreting guild‐, species‐, and individual‐level trophic strategies. Reef fish isotope compositions varied significantly among, but also within, guilds. Individuals of the same species did not cluster together based on their isotope values, suggesting within‐species specializations. Furthermore, while two‐dimensional isotopic niches helped differentiate reef fish resource use, niche overlap among species was exceptionally high. The addition of *δ* ^34^S and the generation of three‐dimensional isotopic niches were needed to further characterize their isotopic niches and better evaluate potential trophic strategies. These data suggest that *δ* ^34^S may reveal fluctuations in resource availability, which are not detectable using only *δ* ^15^N and *δ* ^13^C. We recommend that researchers include *δ* ^34^S in future aquatic food web studies.

## Graphical abstract

We use Stable Isotope Bayesian Ellipses in R (SIBER) to investigate cross‐guild trophodynamics of reef fish species within an oceanic atoll. We compared two‐ (δ13C and δ15N) versus three‐dimensional (δ13C, δ15N, and δ34S) reconstructions of isotopic niche space for interpreting guild‐, species‐, and individual‐level trophic strategies. Reef fish isotope compositions varied significantly among, but also within, guilds, and while two‐dimensional isotopic niches helped differentiate reef fish resource use, niche overlap among species was exceptionally high. The addition of δ34S and the generation of three‐dimensional isotopic niches was needed to further characterize their isotopic niches and better evaluate potential trophic strategies; these data suggest that δ34S may reveal fluctuations in resource availability which are not detectable using only δ13C and δ15N.

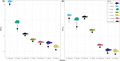

## 1 INTRODUCTION

Characterizing the dietary variation of individuals within a larger population is essential for delineating a species' trophic niche (Bolnick et al., 2003). Yet, at the species and sometimes trophic guild level (i.e., similar trophic strategy), it is frequently assumed that co‐occurring individuals are ecologically equivalent; that is, that species‐level analyses are sufficient for exploring and understanding food web dynamics and energy flows (Bolnick et al., 2003). However, individual specialization within species is widespread (Araújo et al., 2011; Bolnick et al., 2002; Nalley, Donahue, Heenan, & Toonen, 2022; Wyatt et al., 2019). In fact, among all taxa documented, fish have the highest occurrences of individual specialization (Araújo et al., 2011). Their niches vary due to individual behavior and preferences, as well as environmental‐ (Sánchez‐Hernández et al., 2021), and population‐specific (Svanbäck & Persson, 2004) drivers. It is important to identify these inter‐ and intraspecific specializations as they have implications for the ecological roles that the organisms may play (Des Roches et al., 2018). Similarly, determining the degree of trophic plasticity within species will help understand how they may fare against environmental change. Species‐level analyses may, therefore, mask individual differences in dietary variation and limit the ability to identify more complex energy flows and trophodynamics within a specific study area or differences in species' ecological roles. For example, using DNA metabarcoding, Nalley, Donahue, and Toonen (2022) showed that the convict surgeonfish (*Acanthurus triostegus*) had little variability in diet composition among individuals at a site, but that diet composition varied among sites. In contrast, the conspecific brown surgeonfish (*A. nigrofuscus*) exhibited greater variation in diet among individuals, but individuals had a similar diet composition across all sites. However, while such studies can resolve species‐level differences in prey, they are also limited to the gut contents of the animal at the time of sampling (Nalley, Donahue, Heenan, & Toonen, 2022). Like metabarcoding, isotope tracer techniques using multiple isotopes are another tool that can capture individual‐level dietary variation, improving trophodynamic assessments.

Stable isotope analysis (SIA) can explore individual and group trophodynamics through the construction of isotope niches (Newsome et al., 2007). Unlike metabarcoding, the stable isotope ratios obtained from an organism's tissues reveal “time‐integrated averages” of dietary information about the species in question (Hutchinson, 1978; Newsome et al., 2007), giving insight into their trophic ecology (Boecklen et al., 2011). Trophic niche studies that use stable isotopes frequently use the heavy/light isotope ratios of nitrogen ^15^N/^14^N (represented in delta notation as *δ* ^15^N) and carbon ^13^C/^12^C (*δ* ^13^C) to determine wider food web structure and functioning (Layman et al., 2007; Perkins et al., 2014). Accurate interpretations rely on isotopically distinct food sources, so SIA works best in systems where either, or both, *δ* ^15^N and *δ* ^13^C values for resources are well separated. When applied to a large enough sample set, SIA can quantify resource assimilation at both the individual (Fox et al., 2019) and group (McMahon et al., 2016 and sources contained within) level. Quantification using Bayesian analytical methods, such as Stable Isotope Bayesian Ellipses in R (SIBER; Jackson et al., 2011), has rapidly grown in popularity (Shipley & Matich, 2020; Skinner et al., 2022) because of their ability to statistically quantify niche space (Jackson et al., 2011). SIBER aims to explain population variability and has quantified the isotopic niches of a diverse array of aquatic consumers from mobile species such as tuna (Laiz‐Carrión et al., 2019), to sessile corals (Conti‐Jerpe et al., 2020; Price et al., 2021; Santos et al., 2021; Wall et al., 2021) and sponges (Freeman et al., 2015; Shih et al., 2020), benthic fish and crustaceans (Ponce et al., 2021), reef fish in the Caribbean (Stuthmann & Castellanos‐Galindo, 2020), and damselfish in the Pacific (Gajdzik et al., 2018). However, within‐species individual specialization and/or overlapping isotope values in dietary sources make reliance on only *δ* ^15^N and *δ* ^13^C for isotopic niche characterization unsuitable in certain scenarios. In these cases, additional information (i.e., ecological processes of resource partitioning) or advanced stable isotope techniques may be needed to accurately determine how individual variation affects a species' wider ecological role (Matich et al., 2021).

Including additional dimensions to SIA that are ecologically relevant offers one solution to increase understanding of isotopic (and potentially trophic) variability. For example, recent work by Besnard et al. (2021; *δ* ^15^N, *δ* ^13^C, and *δ* ^202^Hg isotopes) and Skinner, Mill, et al. (2019; *δ* ^15^N, *δ* ^13^C, and *δ* ^34^S isotopes) has shown the utility of three‐dimensional SIBER to investigate complex trophic strategies where two dimensions have failed. This three‐dimensional method successfully differentiated among pelagic shark trophic strategies that did not vary significantly by resource use (*δ* ^15^N and *δ* ^13^C) but were characterized by depth gradients (additional *δ* ^202^Hg isotope; Besnard et al., 2021), and revealed within‐species individual specializations in feeding strategies of teleost reef fish that were not detectable using just *δ* ^15^N and *δ* ^13^C (additional *δ* ^34^S isotope; Skinner, Mill, et al., 2019). Incorporating additional isotopic information is relatively simple with the advancement of SIA instrumentation, yet examples of its utility across different environments are limited. Moreover, both two‐ and three‐dimensional isotopic niche studies have mostly focused on detecting niche variation among similar species (Espinoza et al., 2019; Frisch et al., 2014, 2016; Matich et al., 2011; Shipley et al., 2019; Skinner, Mill, et al., 2019). This is likely due to the seemingly similar functional roles of many species, which raises questions as to their ability to co‐exist and share resources. Yet, aquatic systems are complex, with a myriad of trophic relationships that exist outside of related trophic guilds. Additional examples of three‐dimensional SIBER that explore cross‐guild relationships are needed.

Here, we investigated the utility of three‐dimensional SIBER analysis to identify variation in resource use among 11 sympatric reef fish species that occupy different trophic guilds but a similar average trophic position (Froese & Pauly, 2021). Using *δ* ^15^N, *δ* ^13^C, and *δ* ^34^S, we tested the following null hypotheses across an oceanic atoll lagoon: (1) Reef fish isotopic composition does not differ among species or trophic guilds and there are no relationships with fish length; (2) individuals from the same species and trophic guild will cluster together based on their isotopic compositions; and (3) all reef fish will occupy a similar isotopic space with a high degree of niche overlap, whether using two (*δ* ^15^N, *δ* ^13^C) or three (*δ* ^15^N, *δ* ^13^C, and *δ* ^34^S) isotopes. Finally, we discuss the ecological implications and utility of incorporating a third isotope for future isotopic niche studies.

## 2 METHODS

### 2.1 Site location and context

**FIGURE 1:**
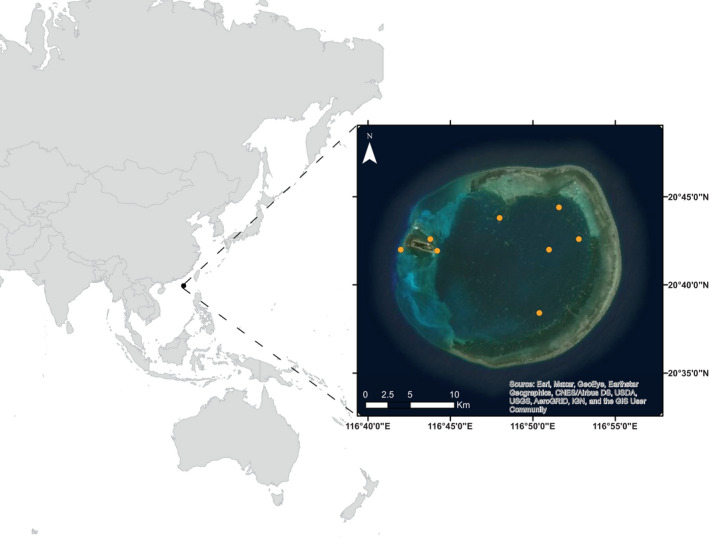
Map of Dongsha Atoll, South China Sea (20°43′N, 116°42′E). Orange dots on the inset indicate areas where reef fish were sampled and the star indicates Pratas Island.

Dongsha Atoll (Dongsha) is the largest and northernmost atoll in the South China Sea (SCS) (20°40′43″ N and 116°42′54″ E; Cheng et al., 2020; Dai, 2005; Figure 1) located 340 km southeast of Hong Kong and 850 km southwest of Taipei. It has a subtropical climate and is influenced by the winter monsoon, with temperatures fluctuating between 28°C in the summer (rainy season) and 20°C in the winter (dry season). The atoll is a reef terrace formed on a seamount. It is approximately 25 km wide, with an inner‐reef and lagoon area of about 600 km^2^ reaching a maximum depth of ~24 m, but generally <12 m (Dai, 2005). The western portion of the atoll is open to the SCS and is where the only associated island (Pratas) is located. The atoll hosts ecologically important coral reef habitats that were established as the Dongsha Atoll National Park (DANP) in 2004 by the Taiwanese government (Cheng et al., 2020).

Ecologically, the inner lagoon area is characterized by highly diverse patch reefs dominated primarily by reef corals from the *Porites* and *Acropora* genera (Cheng et al., 2020; Dai et al., 2013; Xu et al., 2021). Large seagrass beds, composed mostly of *Thalassia hemprichii*, encompass Pratas Island and extend to the northern and southern tips of the atoll crest (Dai, 2005). These coral and seagrass habitats host a high diversity of widely distributed reef fish species (Shao et al., 2011), most of which are classified as grazers (Lee et al., 2014, 2015, 2019).

Oceanographically, Dongsha has currents that generally flow east‐to‐west, but forereef hydrodynamics are strongly influenced by internal waves (IW; Alford et al., 2015; Davis et al., 2020). Although IW mainly affect the outer reef slope, their influence may also reach the atoll lagoon itself. These IW originate from the Luzon Straight (Davis et al., 2020), bringing deeper ocean water and associated nutrients to the atoll (Reid et al., 2019). On average, two waves occur every hour, though they range in timing and intensity (Davis et al., 2020). Typical waves drive water temperature fluctuations between 1 and 4°C (Reid et al., 2019), but larger and less frequent waves can create temperature fluctuations up to 10°C (Davis et al., 2020). Besides changing the thermal environment, IW increase the relative influence of deeper pelagic particulate organic matter (POM) to the Dongsha Atoll environment (Wang, 2016). This addition of deeper pelagic POM to the atoll modulates nutrient concentrations and leads to enhanced biological productivity (DeCarlo et al., 2015).

The lagoon at Dongsha is a useful setting to explore the utility of multiple isotopes in better understanding reef fish trophodynamics. First, it is distant from major human developments and has less direct human influences than many reef habitats (Dai, 2005); at any time, there are a maximum of about 200 people living on Pratas Island. Second, the physical boundary of the lagoon limits cross‐reef‐crest movements reducing the potential for reef fish to access non‐lagoonal resources. While ontogenetic movements across lagoon boundaries and corresponding shifts in resource use have been recorded in other locations (Dale et al., 2011), the reef fish sampled here have small home ranges (Green et al., 2015) and were predominantly adults. As such, although we assume that there are no foraging or ontogenetic movements outside the lagoon boundaries for these individuals, we nonetheless explored fish length—isotope relationships in our analyses. Third, because of its small area, environmental conditions and shifts in isotopic baselines of the food web should affect all species equally. Finally, although our focal species are from different trophic guilds, they occupy similar trophic positions. This allows exploration of inter‐ and intra‐guild reef fish trophodynamics and the utility of a three‐dimensional SIA when environmental conditions are consistent but there is a wide pool of available resources.

### 2.2 Sampling

We selected eleven reef fishes as target species based on their common occurrence at Dongsha Atoll and their range of trophic guilds (Froese & Pauly, 2021): detritivore *Ctenochaetus striatus n* = 4 (McMahon et al., 2016); herbivore *Acanthurus nigricans n* = 15 (Choat et al., 2002, 2004); invertivore *Halichoeres hortulanus n* = 14 (Kramer et al., 2016); omnivores *Amphiprion clarkii n* = 14, *Chaetodon auriga n* = 30, and *Dascyllus aruanus n* = 16 (Frédérich et al., 2009; Nagelkerken et al., 2009; Sano et al., 1984); pisci‐invertivores *Lutjanus fulvus n* = 3, *Lutjanus kasmira n* = 6, and *Neoniphon sammara n* = 20 (DeFelice & Parrish, 2003; Hobson, 1974); and planktivores *Chromis viridis n* = 18 and *Myripristis berndti n* = 5 (Hobson, 1974; Wyatt et al., 2012). In March 2017, tissue samples for these eleven species were collected by SCUBA divers using spears (Figure 1). All fish were collected at depths of 2–10 m. The total length (cm) of each fish was measured, and samples of white muscle tissue (~1 g) were taken from behind the pectoral fin. Tissues were then oven dried at 50°C and stored in 2‐ml microcentrifuge tubes with desiccator beads. Sampling was conducted under permit number 0000691 approved by the Marine National Park Headquarters in Taiwan.

Primary producers and benthic feeders were sampled to constrain potential food sources (Table S1). Thirteen genera of hard corals and their associated symbionts (*n* = 291), as well as one species of gorgonian (*n* = 2), were collected from the same sites as fish to represent benthic consumers. Seagrass (*n* = 5) was collected from around Pratas Island. Three horizontal plankton tows at 330 μm were conducted crossing the center of the lagoon. Additionally, surface water from the same sites as the tows was first sieved at 330 μm and then filtered through a Whatman GF/F filter (0.7 μm) to collect particulate organic matter (POM; *n* = 1). All tissues were oven dried at 50°C and stored with desiccator beads until analysis. Sampling was conducted under permit number 1060000692, approved by the Marine National Park Headquarters in Taiwan.

### 2.3 Isotope selection

Sulfur (*δ* ^34^S) was chosen as an additional isotope to nitrogen (*δ* ^15^N) and carbon (*δ* ^13^C) for both analytical and ecological reasons. First, *δ* ^34^S can be measured simultaneously with *δ* ^15^N and *δ* ^13^C. Second, *δ* ^34^S presents distinct values for different water sources (Peterson & Fry, 1987) and aquatic primary producers (Connolly et al., 2004), with less trophic discrimination than nitrogen (McCutchan et al., 2003). In a study on marine food webs, Connolly et al. (2004) concluded that more studies should investigate variation in *δ* ^34^S, particularly where *δ* ^15^N and *δ* ^13^C vary minimally, yet to date, applications of *δ* ^34^S in marine food web studies remain rare (Skinner et al., 2022). The Dongsha lagoon is oceanic with no freshwater and little terrestrial inputs (Dai, 2005) and minimal human influence (Ren et al., 2017). As such, we assume fluctuations in *δ* ^34^S values should primarily be due to dietary source variation and unrelated to water sources or human influences. We assume this to be true for all our focal reef fish species as we did not sample any higher trophic level predators that might carry out cross‐habitat movements or feed outside the lagoonal boundary.

### 2.4 Stable isotope analysis

Dried muscle tissue was homogenized with a mortar and pestle and then lipid extracted using a modified Folch technique (Jordi et al., 1957). In short, homogenized samples were soaked in Folch solution (2:1 chloroform: methanol) for 2 h. Lipid‐extracted tissue was then dried, weighed to approximately 2 mg in 3 × 5 mm tin capsules, and analyzed for *δ* ^15^N, *δ* ^13^C, and *δ* ^34^S values (NCS‐SIA).

NCS‐SIA was carried out at the Stable Isotope Ratio Mass Spectrometry Laboratory at the University of Hong Kong in July 2021. Samples were combusted in an Elemental Analyser (EA) IsoLink system (Thermo Scientific, Germany) at 1020°C. Helium was used as the carried gas and brought the subsequently produced N_2_, CO_2_, and SO_2_ to a coupled Delta V Advantage isotope ratio mass spectrometer (IRMS; Thermo Scientific, Germany). Stable isotope ratios are reported using the delta (*δ*) notation which for *δ* ^15^N, *δ* ^13^C, and *δ* ^34^S is: [(Rsample∕Rstandard) − 1], where R is the ratio of the heavy to light isotope (e.g., ^13^C/^12^C), and measured values are expressed in per mil (‰).

International reference materials (*n* = 4 per run) were placed at the start and end of each analysis (~60 samples) to correct the isotope values. Reference materials were glutamic acid USGS‐40 (analytical precision, SD: *δ* ^15^N = 0.22; *δ* ^13^C = 0.09) and USGS‐41a (analytical precision, SD: *δ* ^15^N = 0.32; *δ* ^13^C = 0.15) for *δ* ^15^N and *δ* ^13^C, and silver sulfide standards IAEA‐S1 and S2 for *δ* ^34^S (analytical precision, SD: 0.25 and 0.19, respectively). USGS‐42 (analytical precision, SD: *δ* ^15^N = 0.22; *δ* ^13^C = 0.13; *δ* ^34^S = 0.18) was analyzed every six samples and used as an internal analytical standard for drift correction. Blanks (crushed empty tin capsules) were run every three samples.

### 2.5 Data analysis

All analyses were carried out in R 4.1.0 interfaced with RStudio 1.4.1717 (R Core Team, 2021). Species with samples <5 (i.e., *Ctenochaetus striatus* and *Lutjanus fulvus*) were removed prior to statistical analyses. Differences in the composition of individual isotopes among the remaining nine species were initially tested by running generalized linear models (GLM) with either *δ* ^15^N, *δ* ^13^C, or *δ* ^34^S as the response variable and species as a predictor variable. ANOVA *F*‐tests using Satterthwaite's degrees of freedom determined whether the overall effect of species was significant. To determine whether isotopic composition varied among species within the same trophic guild, Tukey post hoc pairwise comparisons were calculated for each species pair using the R package multcomp v1.4–15 (Hothorn et al., 2008). For each species, relationships between fish length (cm) and *δ* ^15^N, *δ* ^13^C, or *δ* ^34^S were explored using a separate linear regression for each isotope (i.e., *y* ~ *x*, where the isotope is the response variable and fish length is the predictor variable).

Univariate tests highlight differences in individual isotopes, but they do not take into account all dimensions, which is important when investigating isotopic niches. To determine how isotopic composition varied among species when considering all three isotopes, we ran a Euclidean Permanova with 999 permutations using the vegan v2.5–7 package (Oksanen et al., 2020). Post hoc pairwise comparisons between species belonging to the same trophic guild were carried out using the PairwiseAdonis package (Martinez Arbizu, 2017).

Before further analysis, all species with samples <10 were removed (leaving seven species from the original 11). Individual variation in feeding strategies (or metabolism or growth) means that fish from the same species, or trophic guild, may not always have similar isotope values. To determine the degree of similarity in isotopic composition among individuals of the same species, we used Ward's hierarchical clustering based on Euclidean distance of *δ* ^15^N, *δ* ^13^C, and *δ* ^34^S values. Clustering was conducted using the factoextra v1.0.7 package in R (Kassambara & Mundt, 2020). The number of clusters was first set to seven to determine if fish clustered by species and then set to five to determine if they clustered instead by trophic guild.

The isotopic niches of the seven fish species that had *n* > 10 were initially investigated in two dimensions with *δ* ^15^N and *δ* ^13^C using the R package SIBER (Jackson et al., 2011). Isotope data are presented on a bi‐plot and the area (*δ*‐space) of the coordinates represents the animal's isotopic niche, with the size and position of the ellipses surrounding the individual coordinates reflecting some aspects of the animal's trophic niche (Bearhop et al., 2004; Newsome et al., 2007). Using SIBER, the 95% Bayesian Standard Ellipse Area (SEA_B_) was calculated for each ellipse for each species (20,000 iterations, burn‐in 1000, thin 10). When sample sizes are small (*n* < 20), it is recommended that standard ellipses be corrected for this (denoted SEA_c_). However, SEA_B_ effectively captures the properties of SEA_c_ and is unbiased to sample size, while also reflecting the uncertainty associated with smaller sample sizes (Jackson et al., 2011). The degree of niche overlap among species was calculated based on these 95% SEA_B_ ellipses. The overlap was expressed as a percentage of the sum of the non‐overlapping area of the ellipses for each species, *a*:

(1) $$\frac{{\text{Overlap}}_{a}}{{\mathrm{SEA}}_{B_{a}}}\;x\;100$$

where 100% indicates completely overlapping ellipses and 0% indicates entirely distinct ellipses. When the overlap in shared isotopic space between two species was >60%, it was considered to be significant (Matley et al., 2017).

To extend the isotopic niche to three dimensions and include *δ* ^34^S, Bayesian ellipsoids were fit to 95% of the data (SEV_B_) (15,000 iterations, burn‐in 10,000, thin 25) and their median volume was determined (Skinner, Mill, et al., 2019). As with the ellipses, SEV_B_ reflects the properties of small sample size corrected ellipsoids (SEV_c_) and slightly underestimates SEV when sample sizes are smaller (Skinner, Mill, et al., 2019). Ellipsoid overlap among species was calculated based on Bayesian SEV_B_ (7500 iterations, burn‐in 5000, subdivision 4). Overlap was expressed as a median percentage where 100% indicates completely overlapping ellipsoids and 0% indicates entirely distinct ellipsoids. When ellipsoid overlap between two species was >60%, it was considered significant (Matley et al., 2017; Skinner, Mill, et al., 2019). To compare the size of the ellipse areas with the ellipsoid volumes for each species, SEA_B_ and SEV_B_ values and their interquartile ranges (2.5%–97.5%) were mean‐centered and scaled using the base functions in R.

## 3 RESULTS

**FIGURE 2:**
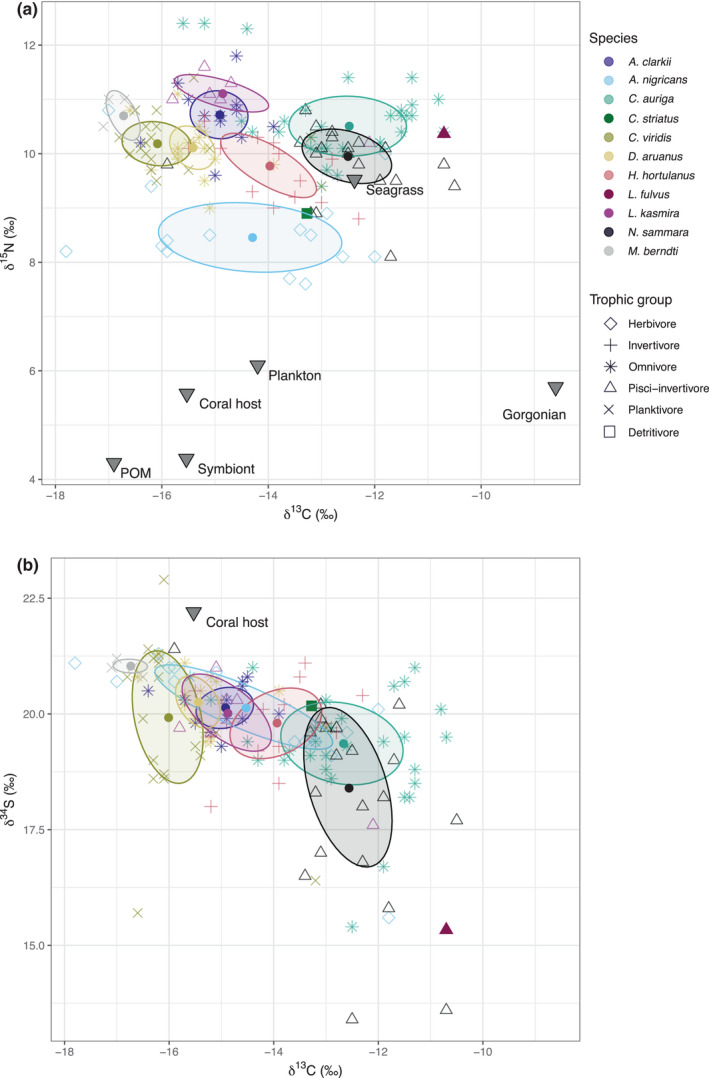
Isotopic biplots of (a) *δ* ^13^C and *δ* ^15^N and (b) *δ* ^13^C and *δ* ^34^S biplots for all 11 species from six trophic guilds sampled across Dongsha Atoll lagoon. Points are individual fish samples, and ellipses are 40% ellipses with centroids. Note that due to small sample sizes, detritivore *C. striatus* (*n* = 4) and pisci‐invertivore *L. fulvus* (*n* = 3) are plotted only as means (filled in shapes). Potential food sources are plotted as filled in downwards triangles with values derived from unpublished data from authors. Note that for *δ* ^34^S, only coral host data are available for the potential food sources.

In total, 143 samples were collected from 11 reef fish species across six trophic guilds to understand fish community dynamics (Table 1; Figure 2). However, only fish species with samples *n* > 5 were included in statistical analyses, leaving 136 samples from nine species belonging to five trophic guilds. Although *C. striatus* (detritivore; *n* = 4) and *L. fulvus* (pisci‐invertivore; *n* = 3) were not analyzed statistically, their incorporation in this manuscript aims to bolster known datasets for these species which are currently sparse. Furthermore, their position in isotopic space can help give context to other species, as *L. fulvus* is the most enriched in *δ* ^13^C of any species, and *C. striatus* is depleted in *δ* ^15^N similar to *C. auriga*.

**TABLE 1:** Summary table of samples taken from eleven reef fish species from six trophic guilds across the lagoon at Dongsha Atoll. Body length (cm) and stable isotope values (*δ* ^15^N, *δ* ^13^C, and *δ* ^34^S) are presented as mean ± standard deviation (SD). *n* = number of samples; range = difference between minimum and maximum values

Species	Trophic guild	*n*	Length (cm)		*δ* ^15^N			*δ* ^13^C			*δ* ^34^S		
			Mean	SD	Mean	SD	Range	Mean	SD	Range	Mean	SD	Range
*A. clarkii*	Omnivore	12	6.71	2.28	10.70	0.55	2.20	−14.98	0.65	2.50	20.14	0.47	1.50
*A. nigricans*	Herbivore	15	13.22	3.05	8.62	0.86	3.20	−14.45	1.92	6.00	19.92	1.42	5.70
*C. auriga*	Omnivore	30	11.58	2.51	10.64	0.74	3.00	−12.69	1.29	4.90	19.31	1.19	5.60
*C. striatus*	Detritivore	4	20.63	2.50	8.90	0.29	0.70	−13.28	0.49	1.20	20.18	0.29	0.70
*C. viridis*	Planktivore	18	5.87	2.09	10.19	0.50	2.00	−15.79	1.07	3.80	19.67	1.78	7.20
*D. aruanus*	Omnivore	16	3.32	2.81	10.09	0.50	2.10	−15.42	0.58	2.70	20.30	0.60	1.90
*H. hortulanus*	Invertivore	14	11.61	2.75	9.77	0.61	1.90	−13.99	0.96	3.20	19.77	0.85	3.10
*L. fulvus*	Pisci‐invertivore	3	20.33	2.36	10.37	0.32	0.60	−10.70	0.70	1.40	15.33	0.93	1.80
*L. kasmira*	Pisci‐invertivore	6	19.58	1.50	11.03	0.47	1.40	−14.63	1.30	3.70	19.75	1.17	3.40
*M. berndti*	Planktivore	5	18.70	2.25	10.60	0.54	1.30	−16.70	0.37	0.90	21.04	0.17	0.40
*N. sammara*	Pisci‐invertivore	20	17.75	2.44	9.82	0.63	2.70	−12.58	1.14	5.40	18.17	2.13	8.00

Among the species retained for analysis, there was a significant difference in *δ* ^15^N (*F* = 16.96; *p* < .001), *δ* ^13^C (*F* = 21.11; *p* < .001), and *δ* ^34^S (*F* = 4.59; *p* < .001; Table S2). Post hoc pairwise comparisons of species within the same trophic guild revealed a significant difference in *δ* ^13^C between the omnivores *A. clarkii* and *C. auriga* and between *C. auriga* and *D. aruanus* (Table S3). Among the pisci‐invertivores, there was a significant difference in *δ* ^15^N and *δ* ^13^C between *L. kasmira* and *N. sammara* and in *δ* ^15^N between the pisci‐invertivore *L. kasmira* and invertivore *H. hortulanus*. There was also a significant difference in *δ* ^13^C and *δ* ^34^S between the pisci‐invertivore *N. sammara* and the invertivore *H. hortulanus*. There were no differences in isotopic composition among the planktivores (Table S3).

There were few significant relationships between fish length (cm) and their isotopic values, and those that were significant (*n* = 4) had low *R* ^2^ (Figure S1). For *δ* ^13^C, there was a significant negative relationship with fish length for *C. auriga* (*F*_1,28_ = 19.13, *R* ^2^ = .41, *p* < .001) and a significant positive relationship for *H. hortulanus* (*F*_1,12_ = 12.12, *R* ^2^ = .50, *p* = .005). There was a marginally significant positive relationship between *δ* ^15^N and length for *A. nigricans* (*F*_1,13_ = 5.10, *R* ^2^ = .28, *p* = .04) and a significant negative relationship between *δ* ^34^S and length for *C. viridis* (*F*_1,16_ = 10.38, *R* ^2^ = .39, *p* = .005) (Figure S1). Relationships between fish length and each isotope were non‐significant for all other species.

**FIGURE 3:**
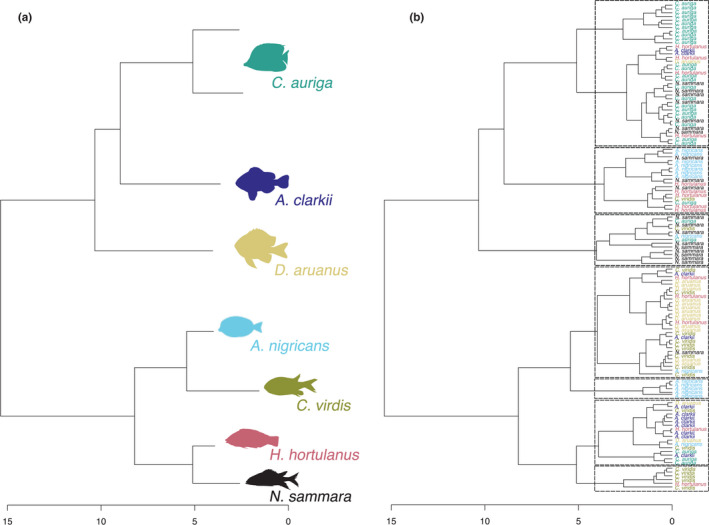
Dendrogram obtained by (a) hypothesized clustering based on ecological theory, and (b) hierarchical cluster analysis of reef fish *δ* ^15^N, *δ* ^13^C, and *δ* ^34^S values (Ward's hierarchical clustering, based on Euclidean distance). The number of clusters was set to *n* = 7 to determine whether fish separated into species‐specific groups. Fish are colored by species.

When considering all three isotopes, there was a significant difference in isotopic composition among species (PERMANOVA, 999 perm, *df* = 10,132, SS = 454.00, *F* = 12.89, *R* ^2^ = .494, *p* < .001). Pairwise post hoc tests determined that all species pairs within each trophic guild were significantly different from one another, except for the planktivores *M. berndti* and *C. viridis* (Table 2). Furthermore, results of hierarchical clustering using the *δ* ^15^N, *δ* ^13^C, and *δ* ^34^S values revealed that fish did not cluster by species or by trophic guild (Figures 3 and S2; Table S4), with individuals from a range of species and trophic guilds co‐occurring across both seven (species; Figure 3) and five (trophic guild; Figure S2) clusters.

**TABLE 2:** Post hoc analysis on a PERMANOVA investigating the effect of species on the isotopic composition (*δ* ^15^N, *δ* ^13^C, *δ* ^34^S) of reef fish across Dongsha Atoll. Pairwise comparisons are carried out on pairs of species belonging to the same trophic guild.

Pairwise comparison	*df*	SS	*F*	*R* ^2^	*p*‐value
**Omnivores**					
*A. clarkii*—*C. auriga*	1	51.215	17.622	.306	**.001**
*A. clarkii*—*D. aruanus*	1	3.992	4.223	.140	**.011**
*C. auriga—D. aruanus*	1	91.336	33.518	.432	**.001**
**Pisci‐invertivores/Invertivores**					
*L. kasmira—N. sammara*	1	37.944	6.746	.219	**.008**
*L. kasmira—H. hortulanus*	1	8.413	3.544	.165	**.031**
*N. sammara—H. hortulanus*	1	37.827	8.341	.207	**.002**
**Planktivores**					
*C. viridis—M. berndti*	1	11.173	2.969	.124	.059

*Note*: Bold and underlined indicates significance at the *p* = .05 level.

Abbreviations: *df*, degrees of freedom; SS, sum of squares.

There were more occurrences of substantial (>60%) isotopic niche overlap for ellipses (*n* = 14; *δ* ^15^N and *δ* ^13^C) than for ellipsoids (*n* = 5; *δ* ^15^N, *δ* ^13^C, *δ* ^34^S; Table 3; Figure S3). Only the ellipse generated for the omnivore *D. aruanus* had no species substantially overlapping with it. Generally, the species with the larger ellipses had more substantial overlaps with other species. Four species had ellipses that substantially overlapped with the ellipse of *A. nigricans*, three species had ellipses that substantially overlapped with the ellipse of *C. auriga* and *C. viridis*, respectively, and two species had ellipses that substantially overlapped with the ellipse of *N*. s*ammara*. When including *δ* ^34^S to generate ellipsoids, there was a >60% reduction in substantial overlaps (Table 3). The only remaining instances of substantial overlap involved the species with the largest niche volumes: *A*. *nigricans*, *C. auriga*, *C*. *viridis*, and *N. sammara*.

**TABLE 3:** Isotopic niche overlaps based on (a) 95% ellipses and (b) 95% ellipsoids. Presented as the proportion (%) of the total area that overlaps. The table is to be read horizontally, that is, for 95% ellipses, *A. clarkii* has a niche that overlaps 52.2% with *A. nigricans*, while the niche of *A. nigricans* overlaps 11.9% with *A. clarkii*.

	A. clarkii	A. nigricans	C. auriga	C. viridis	D. aruanus	H. hortulanus	N. sammara
**(a) 95% ellipses**							
*A. clarkii*	–	52.2	**67.4**	**70.5**	55.3	57.3	43.2
*A. nigricans*	11.9	–	19.8	23.2	13.7	22.7	28.7
*C. auriga*	28.7	36.9	–	20.6	11.2	29.3	**61.0**
*C. viridis*	50.9	**73.2**	35.0	–	51.3	50.4	34.7
*D. aruanus*	**76.2**	**82.4**	36.3	**98.0**	–	**67.8**	38.3
*H. hortulanus*	50.4	**87.5**	**60.6**	**61.4**	43.3	–	**79.6**
*N. sammara*	23.3	**67.8**	**77.4**	26.0	15.0	48.8	–
**(b) 95% Ellipsoids**							
*A. clarkii*	–	42.3	**69.3**	**65.2**	43.3	52.0	47.7
*A. nigricans*	6.5	–	18.7	24.6	9.1	16.9	30.2
*C. auriga*	13.3	22.8	–	20.0	6.7	21.5	55.8
*C. viridis*	15.2	37.0	18.8	–	17.6	24.4	30.8
*D. aruanus*	53.1	**71.6**	42.5	**92.9**	–	54.5	43.5
*H. hortulanus*	27.2	56.5	58.3	56.0	23.4	–	**71.8**
*N. sammara*	7.4	30.1	45.0	20.0	5.6	21.3	–

*Note*: Bold and underlined values indicate significant overlap (>60%).

**FIGURE 4:**
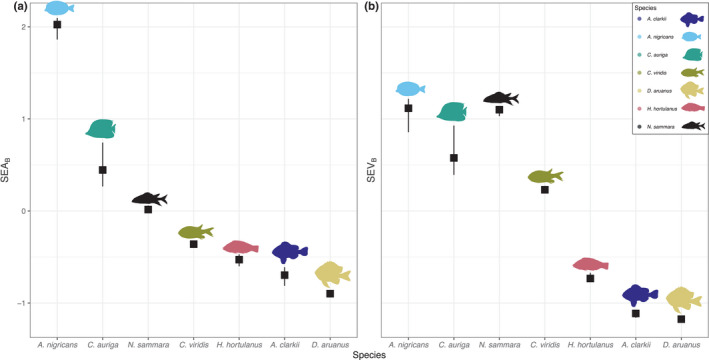
Median ± 95% credible intervals for the (a) 95% Bayesian Standard Ellipse Area (SEA_B_) calculated using *δ* ^13^C and *δ* ^15^N and the (b) 95% Bayesian Standard Ellipsoid Volume (SEV_B_) calculated using *δ* ^15^N, *δ* ^13^C, and *δ* ^34^S for seven fish species sampled across the lagoon at Dongsha Atoll. Values have been scaled and mean‐centered to enable direct comparisons.

The size of the isotopic niches of the seven most‐sampled reef fish (*n* = 125) varied whether they were calculated using *δ* ^34^S or not (Figure 4; Table 4). *A. nigricans* had the largest isotopic niche area regardless of which isotopes were used, although its niche volume decreased with the addition of *δ* ^34^S (Figure 4; Table 4). *N*. *sammara* had the third largest niche using only *δ* ^15^N and *δ* ^13^C, and the second largest isotopic niche when *δ* ^34^S was added. *C*. *viridis* and *C*. *auriga* also had larger isotopic niches when including *δ* ^34^S, but the increase was not substantial. *H*. *hortulanus*, *A*. *clarkii*, and *D*. *aruanus* had the three smallest niches, which were all smaller when incorporating *δ* ^34^S (Figure 4; Table 4).

**TABLE 4:** Bayesian 95% Standard Ellipse Area (SEA_B_) calculated from *δ* ^15^N and *δ* ^13^C and Bayesian 95% Standard Ellipsoid Volume (SEV_B_) calculated using *δ* ^15^N, *δ* ^13^C, and *δ* ^34^S for seven reef fish across Dongsha Atoll. *N* = sample size. Scaled values have been mean‐centered and scaled.

Species	*n*	95% ellipse		95% ellipsoid	
		SEA_B_	SEA_B__Scaled	SEV_B_	SEV_B__Scaled
*A. clarkii*	12	1.17	−0.70	18.78	−1.11
*A. nigricans*	15	5.22	2.02	121.23	1.12
*C. auriga*	30	2.87	0.45	96.44	0.58
*C. viridis*	18	1.67	−0.36	80.55	0.23
*D. aruanus*	16	0.87	−0.90	15.95	−1.18
*H. hortulanus*	14	1.42	−0.53	36.25	−0.73
*N. sammara*	20	2.23	0.02	120.53	1.10

## 4 DISCUSSION

Our study investigated reef fish isotopic ecology across a large and geographically isolated tropical lagoon (Dai, 2005; Dai et al., 2013). We found that reef fish isotopic composition varied substantially across this seascape. The addition of *δ* ^34^S to our analysis substantially decreased the percent overlap of all species' isotopic niches with one another and changed the size of the isotopic niches of several species; it was key in identifying both inter‐ and intraspecific variation in resource use. These findings provide much needed context in terms of the ecology of reef fish in a remote coral reef food web where human influences are low (Dai, 2005; Ren et al., 2017). Furthermore, our findings demonstrate the utility of including *δ* ^34^S in trophic ecology studies. The addition of *δ* ^34^S better highlights dietary variation among individuals and between species, showing it is useful for identifying complex isotopic and trophic ecologies.

### 4.1 The need for multiple dimensions to detect trophic differences among species and guilds

Contrary to our null hypothesis, univariate tests confirmed that all the species differed in their isotopic compositions (*δ* ^15^N, *δ* ^13^C, and *δ* ^34^S). However, pairwise comparisons of species within the same trophic guild were not always different from one another. Although similar isotope values were expected for *δ* ^15^N within each trophic guild, we also found few differences in *δ* ^13^C or *δ* ^34^S. This similarity in isotopic compositions may arise from (i) species within each trophic guild utilizing the same resources or (ii) the isotopic values of their food sources not being distinct. However, multivariate tests analyzing *δ* ^15^N, *δ* ^13^C, and *δ* ^34^S together revealed that not only were species significantly different from one another, but species pairs within the same trophic guild also differed significantly from one another isotopically (except for the planktivores *C. viridis* and *M. berndti*). This suggests that using a single tracer, such as only *δ* ^15^N, *δ* ^13^C, or *δ* ^34^S, may hide important dietary variation, and lead to false conclusions regarding shared resources among groups. Multivariate analyses more accurately capture the extent of the isotopic niche space, representing a more comprehensive view of a species' trophic ecology, which is likely to be inherently variable (Nalley, Donahue, & Toonen, 2022; Skinner, Mill, et al., 2019).

### 4.2 Reef fish do not cluster by species or by trophic guild

Stable isotope studies investigating trophodynamics at the genus‐ or species‐level may miss individual variation within populations, which could be important when trying to characterize a species' trophic ecology. We investigated the variation in the isotopic space of each species through hierarchical clustering of individual fish *δ* ^15^N, *δ* ^13^C, and *δ* ^34^S isotope values. We hypothesized that if individuals within a species used similar food sources, then their isotopic compositions should cluster together or into similar groups. We found the opposite; there was substantial variation in individual isotopic composition and fish did not cluster consistently with their own species (i.e., seven clusters), or even by general trophic guilds (i.e., five clusters). This suggests that the isotopic ranges for several species studied here are likely influenced by individual variation in dietary sources.

There are other possible explanations for the large isotopic ranges and niches of the reef fish though. Firstly, even across small spatial scales, the underlying isotopic baselines can fluctuate. However, these samples were all collected in the same habitat (atoll lagoon) and during the same period (one week), so it is unlikely that individuals of the same species or even the same trophic guild were accessing similar resources with vastly different isotope values. Indeed, our extensive coral sampling across the same sites supports this. Corals and their symbionts can track baseline changes in marine systems as they are supported by inorganic nutrients and organic material at the base of the food web (Wong et al., 2017). In this study, coral isotope values varied minimally across sites and across genera that exhibit diverse trophic strategies (Conti‐Jerpe et al., 2020), and their *δ* ^13^C and *δ* ^15^N values were similar to the algae and POM samples. This consistency suggests that there is minimal spatial variance in the underlying isotopic baselines and thus likely little corresponding influence on the reef fish niches. Secondly, the larger isotopic ranges and niches might be influenced by trophic discrimination differences among species (McCutchan et al., 2003). However, intra‐species trophic discrimination values of assimilated resources are small (<0.8‰) across numerous reef fish encompassing four different guilds (Wyatt et al., 2010) and even smaller (<0.3‰) for omnivores (Britton & Busst, 2018). Lastly, ontogenetic variation in resource use may lead to larger isotopic ranges or niches for some species. However, relationships between isotope ratios and fish body size were mostly absent here, suggesting that differences in resource use are not ontogenetic. Instead, our findings suggest that there are individual‐level specializations occurring which preclude species‐level generalizations from being drawn. This correlates with other work using stable isotopes (e.g., Frédérich et al., 2009; Gajdzik et al., 2016), DNA metabarcoding (e.g., Nalley, Donahue, Heenan, & Toonen, 2022), and gut content analysis both in an experimental setting (Britton & Busst, 2018) and within seagrass habitats across our study site (Lee et al., 2014, 2015, 2019). These studies and interpretations support that several of our focal species exhibit dietary plasticity and can access a diverse range of food types.

A good example of the nuances associated with our interpretations can be seen in *A. clarkii* (omnivore) and *H. hortulanas* (invertivore). Although these two species had the lowest isotopic ranges analyzed, they were still found across three and five clusters, respectively. Often, these two species clustered with those from a different trophic guild altogether. One possible interpretation of this is that they are adjusting to species‐level competition by accessing a diverse range of resources more commonly accessed by other trophic guilds (Bolnick et al., 2007). Moreover, for *H. hortulanus*, there was a significant positive shift in *δ* ^13^C with increasing body size, suggesting that there may be some dietary variation associated with ontogeny, as larger individuals prey on increasingly benthic prey. However, we sampled fish muscle tissue, which has a relatively long tissue‐turnover time of hundreds of days (Boecklen et al., 2011; Winter et al., 2019). Thus, our data are indicative of a long‐term (time‐averaged) reflection of dietary choice for each individual and not a result of a short‐term, opportunistic dietary change. This, therefore, suggests that either both species are predominantly omnivorous or that *A. clarkii* is preying primarily on invertebrates, either to reduce competition or simply because of food availability. Either of these scenarios would lead to these individuals having similar isotopic values across guilds and thus clustering together. Understanding the isotopic ecology of these species, and making inferences about their trophic ecology, is not as straightforward as perceived even within this confined habitat.

### 4.3 Two‐dimensional isotopic niches help explain resource use but indicate substantial overlap

To further explore both inter‐ and intraspecific variation in resource use within the atoll, we generated isotopic niches using SIBER. Generated *δ* ^15^N and *δ* ^13^C niches revealed that the herbivore *A. nigricans* had the largest isotopic niche of all seven of the well‐sampled reef fish. As an herbivore, one could hypothesize that its niche should be smaller compared with omnivores or invertivores, that is, groups that are thought to be accessing a wider range of resources. However, our finding of large niche space in an herbivore is consistent with previous studies using a variety of approaches (Nalley, Donahue, Heenan, & Toonen, 2022). Algal and detrital resources are readily replenished on coral reefs but vary in their accessibility (Bonaldo & Bellwood, 2011; Brandl et al., 2015), but in the Dongsha lagoon, the range of resources available to herbivores is exceptionally diverse due to extensive seagrass beds and associated seagrass‐derived detritus. Furthermore, deeper water during flood tides provides more space for herbivores to forage, potentially expanding available resources (Lee et al., 2014; Lee, 2021). Wide ranges in aquatic plant matter isotope values are also not uncommon, driven by both differential forms of photosynthesis (*δ* ^13^C) and sulfate assimilation (*δ* ^34^S; Peterson et al., 1986; Peterson & Fry, 1987). Similarly, algae δ^13^C values can change in response to light and depth, which may further explain the range in *A. nigricans* δ^13^C values observed here (Wefer & Killingley, 1986; Wiencke & Fischer, 1990). Previous studies have also reported links between *A. nigricans* stable isotope ratios and location‐specific availability of food items (Zgliczynski et al., 2019), with larger isotopic niche widths (particularly along the δ^13^C axis) because of increasing primary production (Miller et al., 2019). Since herbivorous surgeonfish like *A. nigricans* have small home ranges (Green et al., 2015) and access to a wide range of plant material within the lagoon, it is unlikely they leave to forage; their wide range of isotopic values most likely reflect these plants' differing paths for nutrient assimilation and/or microbial reworking of organic material.

Both the omnivore *C. auriga* and the pisci‐invertivore *N. sammara* had the second largest isotopic niches and of a similar size to one another. This is unsurprising for the omnivorous *C. auriga*. Generalist populations are more variable and heterogeneous in their niches (Bolnick et al., 2007) and likely feed opportunistically on a range of available prey items (Nagelkerken et al., 2009). *C. auriga* δ^13^C values also significantly declined with increasing body size, suggesting the wider niche identified here may be driven partly by ontogenetic shifts in diet as they grow larger. Conversely, *N. sammara* is thought to feed predominantly on the zoobenthos such as small crabs and shrimps (Froese & Pauly, 2021). However, like the algal resources, plankton communities within the atoll may have a fluctuating isotopic range due to the prevalence of internal waves sporadically providing deeper water and associated pelagic nutrients (DeCarlo et al., 2015; Reid et al., 2019; Santos et al., 2021). Stable isotope food web studies seldom consider temporal fluctuations in resource availability (Skinner et al., 2022), but this aspect of food web dynamics could explain the larger isotopic range of some of these species. For example, at the same location, hard corals vary in their isotope values temporally as available nutrients fluctuate (Erler et al., 2019; Radice et al., 2019). Consequently, as plankton communities are replenished, these fish may exhibit trophic plasticity (reflected in the large range in their isotope values) by adapting to prevailing environmental conditions (Berg & Ellers, 2010). Nevertheless, future studies would benefit from sampling the underlying plankton and prey communities to better characterize these temporal fluctuations.

While 2‐D isotopic ellipses give a better sense of the trophic strategies employed by the reef fish within the atoll, they also raise further questions. For example, despite supposedly differing resource use (as indicated by the significant differences in individual isotopic ratios), there was a high level of isotopic niche overlap among species both within the same and different trophic guilds. This likely occurs because isotopic niches represent only one aspect of a species' trophic niche and do not necessarily account for spatial partitioning of resources which facilitates coexistence without increasing isotopic niche space. For example, highly diverse pomacentrid assemblages at Dongsha Atoll displayed a similar isotopic niche space to pomacentrids in Moorea (French Polynesia), despite much lower species richness in the latter (Gajdzik et al., 2018). Similarly, and contrary to expectations, surgeonfish with different morphologies have a similar dietary range (Brandl et al., 2015; Nalley, Donahue, & Toonen, 2022), suggesting species may differ across other axes of their trophic niches which are not reflected in the isotopic ratios (Schoener, 1974). It is clear from these examples, and our data, that only analyzing *δ* ^15^N and *δ* ^13^C misses important ecological information that could help explain unexpected niche overlaps. Another method which might be employed when investigating intricate trophic strategies is compound‐specific stable isotope analysis of amino acids, specifically the δ^13^C of essential amino acids termed “δ^13^C fingerprints” (Larsen et al., 2009), which has recently shown promise in differentiating the niches of marine consumers (Larsen et al., 2020).

### 4.4 A three‐dimensional isotope approach reduces niche overlap and reveals dietary variation

The addition of *δ* ^34^S increased the size of the isotopic niches of several species and simultaneously reduced the occurrence of significant niche overlap among species both within and among trophic guilds, a pattern consistent with fish in estuarine systems (Seubert et al., 2019). Following the addition of *δ* ^34^S, while herbivorous *A. nigricans* still had the largest isotopic niche overall, it became similar in size to the niches of the omnivore *C. auriga* and pisci‐invertivore *N. sammara*. *δ* ^34^S is useful for delineating movement, habitat, and diet (Carr et al., 2017; MacAvoy et al., 2017; McCauley et al., 2014), due to the distinct isotope values of sulfate in ocean water, fresh water, sediments, and plant organic material (Peterson & Fry, 1987). In aquatic systems, the addition of *δ* ^34^S can separate fish resource use when *δ* ^13^C cannot (Hesslein et al., 1991; Skinner, Mill, et al., 2019), which could be due to varying dependencies on infaunal invertebrates that reflect reduced microbial isotope values (Kiyashko et al., 2011), organic material derived from plants that assimilate differing sulfate sources (Peterson et al., 1986), differences in marine versus pelagic phytoplankton (Connolly et al., 2004), or all of these sources in some combination (Croisetière et al., 2009). The mean differences and large ranges in *δ* ^34^S values between *A. nigricans, C. auriga*, and *N. sammara* most likely indicate that they are accessing different dietary sources. *N. sammara* is accessing a more depleted food source, likely driven by microbes associated with sediment invertebrates consistent with its pisci‐invertivore diet strategy, while the wide range in *A. nigricans* and *C. auriga* values likely reflect the lagoon's numerous detrital sources. The addition of *δ* ^34^S highlights its importance in characterizing the niches of these species.

Interestingly, the addition of *δ* ^34^S also increased the niche of the planktivore *C. viridis*. In an oceanic atoll in the Maldives, *δ* ^34^S values revealed clear separation between pelagic and reef resources (Skinner, Newman, et al., 2019), and at Palmyra Atoll, Northern Line Islands, *δ* ^34^S was fundamental in separating lagoonal from offshore plankton (McCauley et al., 2014). As *C. viridis* will access and directly benefit from oceanic productivity where possible (Le Bourg et al., 2017; Wyatt et al., 2012), this suggests that the larger *δ* ^34^S ranges here may reflect a plankton community that fluctuates in terms of composition and availability (Santos et al., 2021). Furthermore, there was a significant negative relationship between *C*. *viridis δ* ^34^S values and increasing body size. While this may indicate that this species diversifies its resource use as it grows larger, the absence of a similar relationship with *δ* ^13^C does not support this hypothesis. Instead, *δ* ^34^S can reflect habitat use (McCauley et al., 2014; Skinner, Newman, et al., 2019), indicating there may be a shift from predominantly spending time in the water column to being closer to the substrate as individuals grow larger.

### 4.5 The utility of δ^34^S for aquatic food web and isotopic niche studies

There are now decades of research showing the utility of *δ* ^34^S as an isotope tracer in food web studies (Croisetière et al., 2009; Hesslein et al., 1991; Kiyashko et al., 2011); it is increasingly being recommended as a third isotope to disentangle habitat usage and planktonic resource dependence in coral reef ecosystems (McCauley et al., 2014; Skinner et al., 2022; Skinner, Newman, et al., 2019). Yet, to date, applications of δ^34^S in marine food web studies remain rare and its efficacy in isolated habitats has not been well studied. We tested this in an oceanic atoll with minimal freshwater and human inputs where *δ* ^34^S ranges in consumers should be driven primarily by dietary variation. We show that while *δ* ^15^N and *δ* ^13^C help reveal subtleties of reef fish trophic strategies which differ from their populations elsewhere, *δ* ^34^S is required to get a clearer picture of how these species co‐exist within the atoll lagoon. Even where *δ* ^15^N and *δ* ^13^C values are consistent, an individual's *δ* ^34^S can be highly variable, providing additional context to understanding trophic interactions and competition. This variability is ultimately driven by the combination of organic and inorganic sulfur pools in a consumer's diet, which both have distinct and variable isotope values (McCutchan et al., 2003). Despite a recommendation to utilize *δ* ^34^S in aquatic food web studies almost two decades ago (Connolly et al., 2004), few studies have analyzed it either individually or in combination with *δ* ^15^N and *δ* ^13^C. Given the ease and low cost with which *δ* ^34^S can now be sampled (i.e., from the same sample aliquot as *δ* ^15^N and *δ* ^13^C), we strongly recommend that future aquatic studies employ this three‐dimensional isotope technique to better characterize isotopic variation and inferred trophic strategies within complex food webs.

## AUTHOR CONTRIBUTIONS

**Jonathan David Cybulski:** Conceptualization (lead); data curation (lead); formal analysis (supporting); investigation (equal); methodology (equal); visualization (equal); writing – original draft (lead); writing – review and editing (lead). **Christina Skinner:** Formal analysis (lead); investigation (equal); methodology (equal); software (lead); visualization (equal); writing – original draft (lead); writing – review and editing (lead). **Zhongyue Wan:** Investigation (equal); writing – original draft (supporting); writing – review and editing (equal). **Carmen K.M. Wong:** Data curation (equal); investigation (equal); writing – review and editing (equal). **Rob J. Toonen:** Data curation (equal); resources (equal); writing – review and editing (equal). **Michelle Gaither:** Data curation (equal); resources (equal); writing – review and editing (equal). **Keryea Soong:** Funding acquisition (equal); project administration (supporting); resources (equal); writing – review and editing (supporting). **Alex S.J. Wyatt:** Project administration (supporting); supervision (supporting); writing – review and editing (equal). **David M. Baker:** Conceptualization (equal); data curation (equal); funding acquisition (lead); writing – review and editing (equal).

## CONFLICT OF INTEREST

The authors have declared no conflicts of interest for this article.

### OPEN RESEARCH BADGE

This article has earned an Open Data badge for making publicly available the digitally‐shareable data necessary to reproduce the reported results. The data is available at: https://github.com/joncybulski/DongshaFishSIA.

## Supporting information


**Appendix S1** Supporting Information

## Data Availability

The data that support the findings of this study are openly available in Supporting Information or at https://github.com/joncybulski/DongshaFishSIA.
