# Supplementary material for: Improving stable isotope assessments of inter‐ and intra‐species variation in coral reef fish trophic strategies
Source: Ecol Evol. 2022 Sep 13;12(9):e9221. doi: 10.1002/ece3.9221 (PMC9468908; doi:10.1002/ece3.9221)
Supplement: Supplementary file 1 — Appendix S1 Supporting Information [file ECE3-12-e9221-s001.docx]

**Improving stable isotope assessments of inter- and intra-species variation in coral reef fish trophic strategies**

# Supplementary


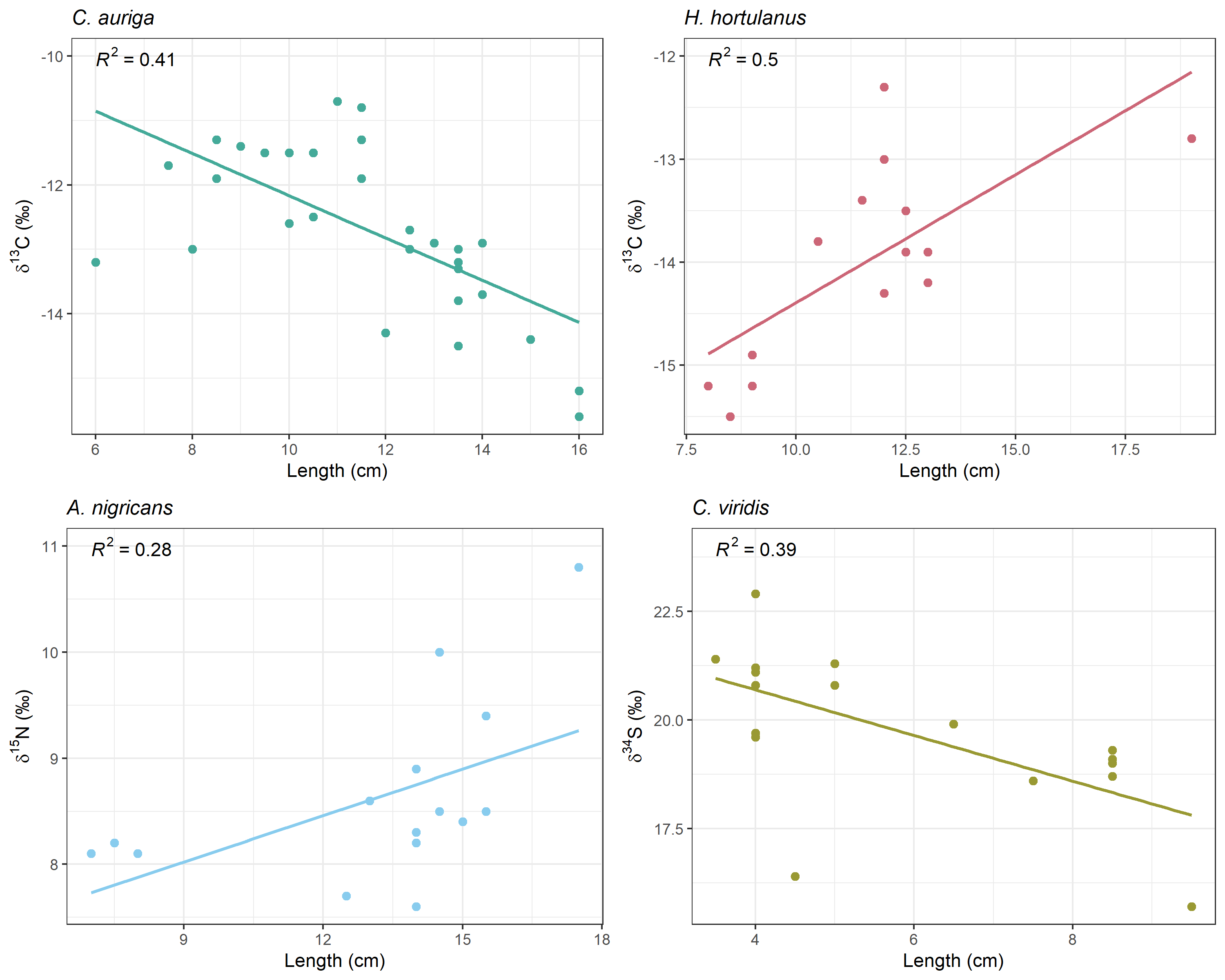


**Fig. S1.** Significant relationships between fish length (cm) and δ^13^C, δ^15^N, or δ^34^S for four reef fish species identified through linear regressions. R^2^ values are displayed on each plot. Relationships between fish length (cm) and each isotope were non-significant for all other reef fish species.


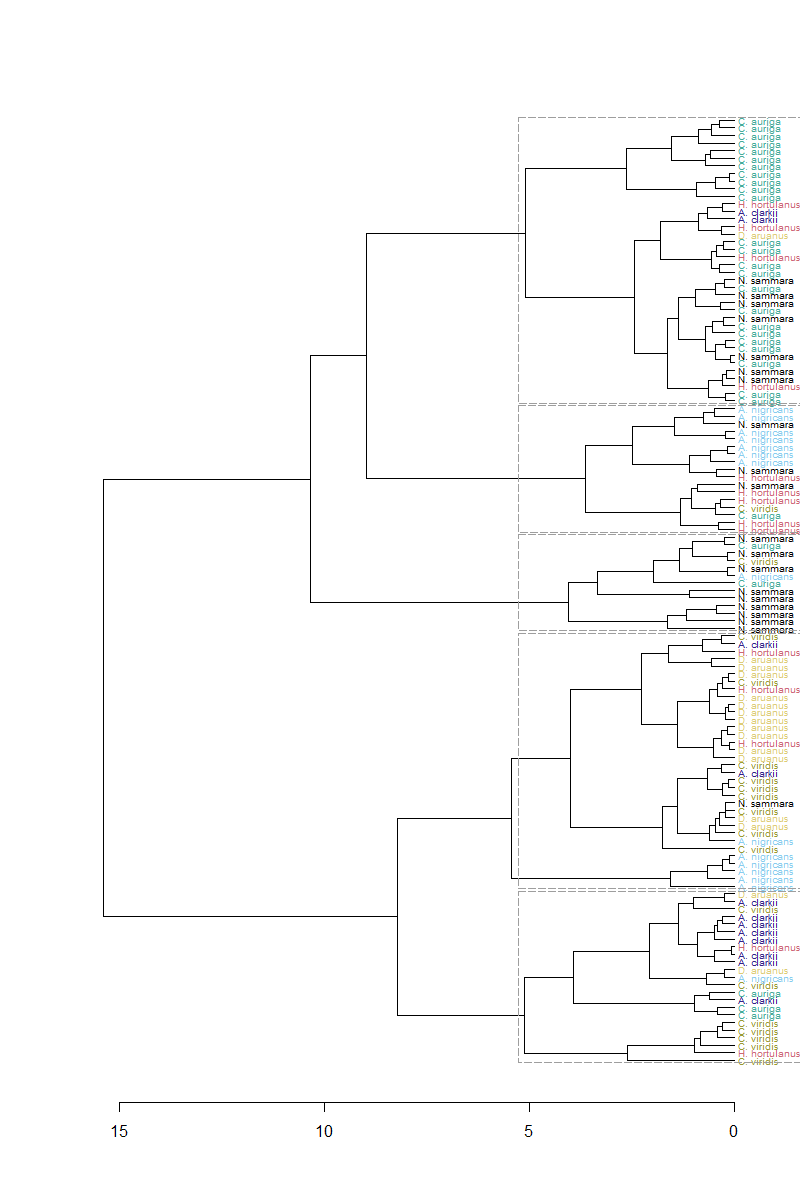


**Fig. S2.** Dendrogram obtained by hierarchical cluster analysis (Ward’s hierarchical clustering, based on Euclidean distance) on reef fish *δ*^15^N, *δ*^13^C, and *δ*^34^S. The number of clusters was set to n = 5 to determine whether fish separated into the five different trophic guilds.

**Fig. S3.** Reef fish isotopic niches displayed as **A)** 95% Bayesian Standard Ellipse Area (SEA_B_) generated using *δ*^15^N and *δ*^13^C and **B)** 95% Bayesian Standard Ellipsoid Volume (SEV_B_) generated using *δ*^15^N, *δ*^13^C, and *δ*^34^S.

**Table S1.** Summary of isotope data for benthic feeders and primary producers collected around Dongsha Atoll to help characterize potential food sources for sampled fish. SD = standard deviation. Standard errors are in brackets after SD for coral samples only. Sulfur isotope data are only available for coral hosts.

|  |  |  | ***δ*^13^C** | | ***δ*^15^N** | | ***δ*^34^S** | |
| --- | --- | --- | --- | --- | --- | --- | --- | --- |
| ***Taxa*** | ***Species*** | ***n*** | ***Mean*** | ***SD*** | ***Mean*** | ***SD*** | ***Mean*** | ***SD*** |
| Coral host | * | 291 | -15.5 | 1.9 (0.1) | 5.6 | 2.2 (0.1) | 22.2 | 1.2 |
| Symbiont | * | 291 | -15.5 | 2.1 (0.1) | 4.4 | 1.1 (0.1) | - | - |
| Gorgonian | Unknown sp. | 2 | -8.6 | 0.2 | 5.7 | 0.1 | - | - |
| Seagrass | *Cymodocea* | 5 | -12.4 | 0.6 | 9.5 | 0.6 | - | - |
| Plankton | > 330 µm | 3 | -14.2 | 0.4 | 6.1 | 0.2 | - | - |
| POM | > 0.7, < 330 µm | 1 | -16.9 | - | 4.3 | - | - | - |

* Coral data is a composite of sampling 13 different genera: *Acropora* (n = 53), *Favites* (n = 44), *Fungia* ( n = 11), *Echinophyllia* (n = 2), *Galaxea* (n = 23), *Goniastrea* (n = 10), *Goniopora* (n = 26), *Hydnophora* (n = 10), *Montipora* (n = 2), *Pavona* (n = 19), *Pocillopora* (n = 21), *Porites* (n = 41), and *Turbinaria* (n = 29). SD = standard deviation.

|  | ***δ*^13^C** | | | ***δ*^15^N** | | | ***δ*^34^S** | | |
| --- | --- | --- | --- | --- | --- | --- | --- | --- | --- |
| *Predictors* | *Estimates* | *CI* | *p* | *Estimates* | *CI* | *p* | *Estimates* | *CI* | *p* |
| (Intercept) | -15.42 | -15.99 – -14.84 | **<0.001** | 10.09 | 9.78 – 10.41 | **<0.001** | 20.30 | 19.63 – 20.97 | **<0.001** |
| *A. clarkii* | 0.44 | -0.44 – 1.31 | 0.333 | 0.61 | 0.13 – 1.09 | **0.015** | -0.16 | -1.17 – 0.86 | 0.761 |
| *A. nigricans* | 0.97 | 0.15 – 1.80 | **0.023** | -1.47 | -1.93 – -1.02 | **<0.001** | -0.38 | -1.34 – 0.58 | 0.437 |
| *C. auriga* | 2.73 | 2.02 – 3.44 | **<0.001** | 0.55 | 0.16 – 0.94 | **0.006** | -0.99 | -1.82 – -0.17 | **0.020** |
| *C. viridis* | -0.38 | -1.17 – 0.41 | 0.353 | 0.10 | -0.33 – 0.53 | 0.648 | -0.63 | -1.54 – 0.29 | 0.181 |
| *H. hortulanus* | 1.43 | 0.58 – 2.27 | **0.001** | -0.32 | -0.78 – 0.14 | 0.172 | -0.53 | -1.50 – 0.45 | 0.289 |
| *L. kasmira* | 0.79 | -0.31 – 1.89 | 0.164 | 0.94 | 0.34 – 1.54 | **0.003** | -0.55 | -1.82 – 0.72 | 0.399 |
| *M. berndti* | -1.28 | -2.46 – -0.10 | **0.035** | 0.51 | -0.14 – 1.15 | 0.126 | 0.74 | -0.62 – 2.10 | 0.289 |
| *N. sammara* | 2.84 | 2.07 – 3.61 | **<0.001** | -0.27 | -0.70 – 0.15 | 0.205 | -2.14 | -3.03 – -1.24 | **<0.001** |
| Observations | 136 | | | 136 | | | 136 | | |
| R^2^ | 0.571 | | | 0.517 | | | 0.224 | | |

**Table S2.** Summary output for GLMs investigating the effect of reef fish species on *δ*^13^C, *δ*^15^N, and *δ*^34^S. CI = confidence intervals; p = p-value. Bold and underlined indicates significance at the p = 0.05 level.

**Table S3.** Post-hoc pairwise comparisons for GLMs investigating the effect of reef fish species on *δ*^13^C, *δ*^15^N, and *δ*^34^S. Species belonging to the same trophic guild are compared. Coeff = coefficient; SE = standard error, *Z* = z-value. Bold and underlined indicates significance at the p = 0.05 level.

|  | ***δ*^13^C** | | | | ***δ*^15^N** | | | | ***δ*^34^S** | | | | | |
| --- | --- | --- | --- | --- | --- | --- | --- | --- | --- | --- | --- | --- | --- | --- |
| **Pairwise comparison** | **Coeff** | **SE** | ***Z*** | **P-value** | | **Coeff** | **SE** | ***Z*** | | **P-value** | **Coeff** | **SE** | ***Z*** | **P-value** |
| **Omnivores** |  |  |  |  | |  |  |  | |  |  |  |  |  |
| *A. clarkii* - *C. auriga* | -2.297 | 0.400 | -5.735 | **<0.001** | | 0.057 | 0.219 | 0.259 | | 1.000 | 0.835 | 0.463 | 1.801 | 0.668 |
| *A. clarkii* - *D. aruanus* | 0.435 | 0.448 | 0.972 | 0.987 | | 0.606 | 0.245 | 2.477 | | 0.231 | -0.158 | 0.518 | -0.305 | 1.000 |
| *C. auriga - D. aruanus* | 2.732 | 0.363 | 7.527 | **<0.001** | | 0.550 | 0.198 | 2.770 | | 0.118 | -0.993 | 0.420 | -2.364 | 0.292 |
|  |  |  |  |  | |  |  |  | |  |  |  |  |  |
| **Pisci-invertivores/Invertivores** |  |  |  |  | |  |  |  | |  |  |  |  |  |
| *L. kasmira - N. sammara* | 2.058 | 0.583 | 3.771 | **0.005** | | -1.213 | 0.298 | -4.067 | | **0.001** | -1.585 | 0.632 | -2.509 | 0.217 |
| *L. kasmira - H. hortulanus* | -0.641 | 0.572 | -1.119 | 0.969 | | 1.262 | 0.313 | 4.035 | | **0.002** | -0.021 | 0.662 | -0.032 | 1.000 |
| *N. sammara - H. hortulanus* | 1.418 | 0.409 | 3.470 | **0.014** | | 0.049 | 0.223 | 0.217 | | 1.000 | -1.606 | 0.473 | -3.396 | **0.018** |
|  |  |  |  |  | |  |  |  | |  |  |  |  |  |
| **Planktivores** |  |  |  |  | |  |  |  | |  |  |  |  |  |
| *C. viridis - M. berndti* | 0.906 | 0.593 | 1.528 | 0.834 | | -0.406 | 0.324 | -1.252 | | 0.941 | -1.368 | 0.686 | -1.993 | 0.533 |

**Table S4.** Number of individuals of each species and each trophic guild assigned to each cluster. Clustering was carried out twice: by species (n =7) and by trophic guild (n = 5).

|  | | **Clustering by species** | | | | | | | | | | | | | |  | | **Clustering by guild** | | | | | | | | | |
| --- | --- | --- | --- | --- | --- | --- | --- | --- | --- | --- | --- | --- | --- | --- | --- | --- | --- | --- | --- | --- | --- | --- | --- | --- | --- | --- | --- |
| **Species** | | **1** | | **2** | | **3** | | **4** | | **5** | | **6** | | **7** | |  | | **1** | | **2** | | **3** | | **4** | | **5** | |
| *A. clarkii* | |  | | 8 | |  | | 2 | |  | |  | | 2 | |  | | 8 | | 2 | |  | |  | | 2 | |
| *A. nigricans* | |  | | 1 | | 5 | | 1 | | 1 | | 7 | |  | |  | | 1 | | 6 | | 1 | | 7 | |  | |
| *C. auriga* | |  | | 3 | |  | |  | | 2 | | 1 | | 24 | |  | | 3 | |  | | 2 | | 1 | | 24 | |
| *C. viridis* | | 5 | | 2 | |  | | 9 | | 1 | | 1 | |  | |  | | 7 | | 9 | | 1 | | 1 | |  | |
| *D. aruanus* | |  | | 2 | |  | | 13 | |  | |  | | 1 | |  | | 2 | | 13 | |  | |  | | 1 | |
| *H. hortulanus* | | 1 | | 1 | |  | | 3 | |  | | 5 | | 4 | |  | | 2 | | 3 | | 9 | | 5 | | 4 | |
| *N. sammara* | |  | |  | |  | | 1 | | 9 | | 3 | | 7 | |  | |  | | 1 | |  | | 3 | | 7 | |
| **Guild** | |  | |  | |  | |  | |  | |  | |  | |  | |  | |  | |  | |  | |  | |
| Herbivore | |  | | 1 | | 5 | | 1 | | 1 | | 7 | |  | |  | | 1 | | 6 | | 1 | | 7 | |  | |
| Invertivore | | 1 | | 1 | |  | | 3 | |  | | 5 | | 4 | |  | | 2 | | 3 | | 9 | | 5 | | 4 | |
| Omnivore | |  | | 13 | |  | | 15 | | 2 | | 1 | | 27 | |  | | 13 | | 15 | | 2 | | 1 | | 27 | |
| Pisci-invertivore | |  | |  | |  | | 1 | | 9 | | 3 | | 7 | |  | |  | | 1 | |  | | 3 | | 7 | |
| Planktivore | | 5 | | 2 | |  | | 9 | | 1 | | 1 | |  | |  | | 7 | | 9 | | 1 | | 1 | |  | |
